# Exploring a potential impact of a social marketing campaign on reducing oral cancer incidences in Michigan: an ecological study

**DOI:** 10.1038/bdjopen.2015.5

**Published:** 2015-12-18

**Authors:** Sungwoo Lim, Georgia Spavik, Amid I Ismail

**Affiliations:** 1 Kornberg School of Dentistry, Temple University, Philadelphia, PA, USA; 2 Michigan Cancer Surveillance Program, Michigan Department of Health and Human Services, Lansing, MI, USA

## Abstract

**Objectives/Aims:**

The social marketing campaign was launched in 2005–2007 to address excess risk of oral cancer in Detroit tri-county area, Michigan. We assessed the extent to which the campaign might have contributed to decreasing risk of oral cancer using the modified interrupted time-series design.

**Materials and Methods:**

Oral cancer incidence and mortality data came from Michigan Department of Community Health, and trends of age-adjusted rates during 1990–2009 were compared between intervention and comparison counties via joinpoint regression.

**Results:**

Although mortality rates in both areas decreased in parallel manner, incidence rates decreased during and after the campaign only in the intervention counties. A similar trend was observed in the race-specific analysis. Alternative explanations, including population profiles, health-care access and pre-existing trends, were examined, and these characteristics were comparable between two areas in 1990s and 2000s.

**Conclusions:**

These results suggested that the campaign was more likely to be associated with the decreasing trend of oral cancer incidence in the intervention counties than alternative explanations. Although oral cancer is a deadly disease, its awareness has been relatively low. This study highlights a potential impact of concerted efforts to improve the oral cancer awareness in the high-risk communities.

## Introduction

According to the 1998–2002 Surveillance Epidemiology and End Results data, rate of oral and pharyngeal cancer (‘oral cancer’) incidence among black males living in the Detroit tri-county area (Macomb, Oakland and Wayne) was one of the highest in the United States (25.7 per 100,000 persons).^1^ In 2005–7, the Detroit Oral Cancer Prevention Project, funded by the National Institute of Dental and Craniofacial Research, launched professionally designed multifaceted social marketing campaigns and community outreach programs that primarily targeted black males living in the Detroit tri-county area. During the campaign, 42 billboards, located in the highly visible areas, were used to increase awareness of high risk of oral cancer and promote free screening at a clinic run by the project. In addition, the same message was delivered via 1,327 radio ads during 2 popular radio programs and 2 newspaper ads. For the community outreach, 3 health educators led 242 education sessions across 89 organisations. Last, a toll-free hotline received 1,783 calls, which were largely prompted by the radio ads (57%). A total of 1,020 adults were screened and 78 were referred for further examinations.^2,3^ Dentists and physicians in the area reported increased interest in oral cancer screening by their patients.^2^

Despite evidence for improvement of the oral cancer knowledge in the target area, it is unknown whether the social media campaign has ultimately contributed to reducing risk of oral cancer incidence and mortality. Assessing this impact may not be feasible in a controlled study design because the campaign has targeted a large geographical area. Thus, the purpose of the study was to explore a potential impact of the social marketing campaign on oral cancer incidences and deaths using population-level longitudinal data from the Michigan Cancer Registry and US Census.

## Materials and methods

### Data sources

The primary data used for this analysis were age-adjusted oral cancer incidence and death rates in three intervention and seven comparison counties in Michigan (Berrien, Calhoun, Genesee, Ingham, Jackson, Muskegon and Saginaw) during 1990–2009. These data came from the Division for Vital Records and Health Statistics, Michigan Department of Community Health, which monitors cancer incidences and deaths in Michigan by collecting patient-level information about cancer site and stage at diagnosis and reviewing death certificates. Cancer of oral cavity and pharynx in Michigan Department of Community Health surveillance data was identified according to the *International Classification of Diseases for Oncology*, third edition.^4^ The secondary data were county-level sociodemographic data at 3 time points from Census 2000, American Community Survey 2005 and American Community Survey 2010.^5–7^

### Study variables

A primary dependent variable was age-adjusted rates of oral cancer incidence and mortality in the intervention and comparison counties between 1990 and 2009. Age adjustment was based on the national age distribution from US Census 2000 data. Additional dependent variables included age-adjusted rates of oral cancer incidence by race (white and black) in 1990–2009, and crude rates of oral cancer incidence by five stages (*in situ*, localised, regional, distant and unknown) in the intervention and comparison counties in 1990–2007.

For independent variables, we selected three population characteristics from Census data (% of males, % of blacks and % of people aged 55 years or older) in the intervention and comparison counties because these factors had been associated with oral cancer incidences in previous studies.^8,9^ We examined two additional characteristics: % of unemployed and % of individuals living under the poverty level, because of their association with health-care access such as health insurance and visits to physicians or dentists.

### Statistical analysis

This study adopted interrupted time-series design to examine whether the social marketing campaign affected the oral cancer incidence and mortality rates in the intervention counties. In this design, changes in time series during intervention (e.g., a different regression slope or a different intercept) are interpreted as evidence for an intervention effect. As Shadish *et al.* ^10^ point out, although, causal inference in interrupted time-series design is subject to multiple threats to validity. There may be historic factors that influence outcomes of interest before or during intervention. A single outcome is usually assessed in this design, which may not accurately capture an effect of intervention if it is aimed to influence multiple dimensions such as awareness, knowledge and behaviours (construct validity). In addition, changes in a slope or an intercept may not be immediately observed due to varying levels of diffusion of an intervention effect (delayed causation). To reduce these potential biases, we added some design elements to the original interrupted time-series design. First, we selected seven comparison counties that were similar to the intervention counties in terms of sociodemographic characteristics of the population and had the highest proportion of black residents after the target area. To minimise potential influences from the media campaign, we also ensured that the comparison counties were geographically separated from the intervention counties, and had different media coverage. To address concerns on construct validity, we examined trends of stage-specific oral cancer incidences along with the overall trend. Last, to capture potentially delayed causation, we examined data on outcomes for 2 more years after the end of the intervention.

Trends of sociodemographic characteristics in the intervention and comparison counties were examined to ensure that population profiles were comparable between two areas before and during the intervention. Then, joinpoint regression analyses from National Cancer Institute were conducted to estimate changes in time-series slopes of oral cancer incidence and mortality rates at varying numbers of inflection points.^11^ Monte Carlo permutation tests were carried out to assess whether regression slopes were not different from zero and parallel between the intervention and comparison counties. All statistical analyses were performed using the joinpoint regression program 4.0.4 (Statistical Research and Applications Branch, National Cancer Institute, Calverton, MD, USA). Statistical significance was assumed at *P*<0.05.

## Results

**Figure 1:**
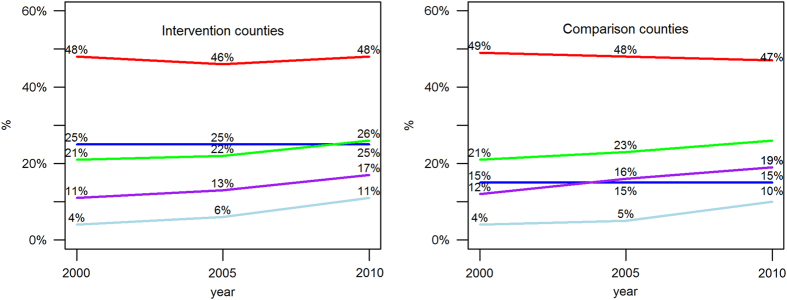
Trends of selected population characteristics in the intervention and comparison counties, Michigan, 2000–2010. Data came from US Census (2000) and American Community Survey (2005, 2010). The intervention included Macomb, Oakland and Wayne counties, whereas the comparison included Berrien, Calhoun, Genesee, Ingham, Jackson, Muskegon and Saginaw counties. Blue line represents % black, red line represents % of male, green line represents % of aged 55 years +, light blue represents % of individuals living<poverty level and purple line represents % of unemployed.

According to Figure 1, key population characteristics in 2000 were comparable between the intervention and comparison counties. An exception was % of blacks, which was much higher in the intervention counties as opposed to the comparison counties (25% vs 15%). Over 10 years, % of individuals aged 55 years or older, % of unemployed and % of individuals living under the poverty level in the two areas increased in parallel manner, whereas % of males and % of blacks remained consistent between 2000 and 2010.

**Figure 2:**
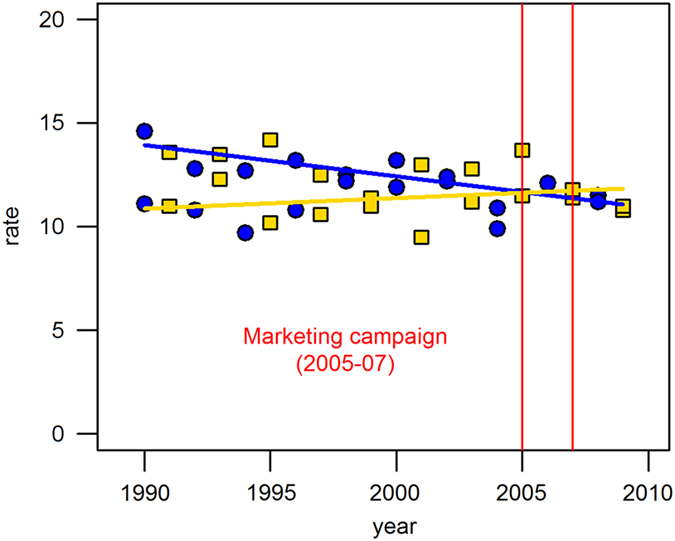
Trends of age-adjusted oral cancer incidence rates per 100,000 persons in the intervention and comparison counties, Michigan, 1990–2009. A blue line represents the trend in the intervention counties (Macomb, Oakland and Wayne counties) and an yellow line represents that in the comparison counties (Berrien, Calhoun, Genesee, Ingham, Jackson, Muskegon and Saginaw). Circle represents intervention counties and square represents comparision counties.

**Figure 3:**
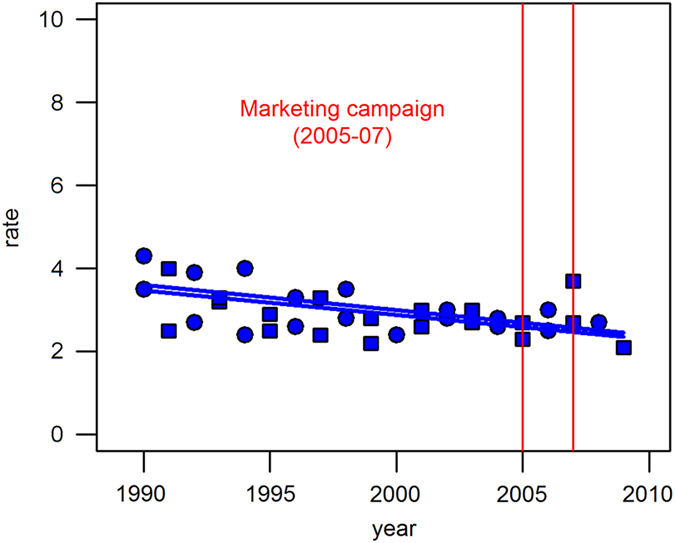
Trends of age-adjusted oral cancer mortality rates per 100,000 Persons in the intervention and comparison counties, Michigan, 1990–2009. The upper line represents the trend in the intervention counties (Macomb, Oakland and Wayne counties) and the lower line represents that in the comparison counties (Berrien, Calhoun, Genesee, Ingham, Jackson, Muskegon and Saginaw). Circle represents intervention counties and square represents comparision counties.

**Figure 4:**
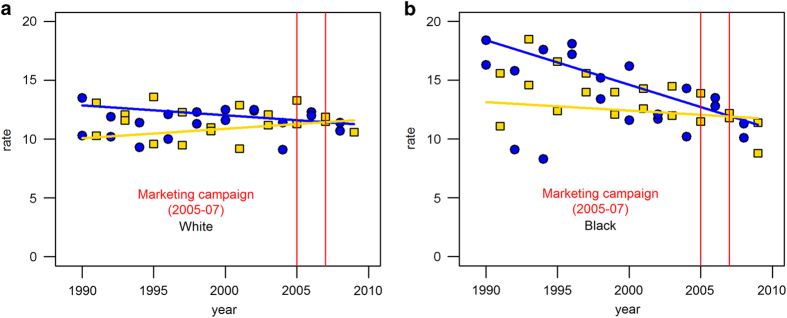
Trends of age-adjusted oral cancer incidence rates per 100,000 persons by race in the intervention and comparison. Counties, Michigan, 1990–2009. (**a**) White: circle represents intervention counties, square represents comparision counties, blue line represents a regression slope for intervention counties and yellow line represents a regression slope for comparison counties. (**b**) Black: circle represents intervention counties, square represents comparision counties, blue line represents a regression slope for intervention counties and yellow line represents a regression slope for comparison counties.

Figure 2 shows that age-adjusted oral cancer incidence rates had decreased between 1990 and 2009 in the intervention counties. Starting from 14 per 100,000 in 1990, the incidence rates annually decreased by 1% on average. In contrast, rates were unchanged in the comparison counties over 20 years. These time series were best described as a straight line (i.e., no inflection point), and the regression slopes were significantly different between the intervention and comparison counties (*P*<0.001). The age-adjusted oral cancer mortality rates decreased by 2% on average and this decreasing trend was parallel between the intervention and comparison counties (Figure 3). A similar trend was observed in the race-specific analysis. The annual rates of oral cancer incidence decreased only in the intervention counties, whereas there was no temporal change in the comparison counties (whites: 1% on average, blacks: 3% on average; Figure 4).

The trend of oral cancer incidence was further examined by stages. Localised and regional diseases represented about 70% of annual oral cancer cases in both areas, and this proportion was consistent during 1990–2009. The annual rate of regional disease increased by 5% on average in the comparison counties, whereas the rate did not change in the intervention counties (Supplementary Figure S1). Similarly, localised disease occurred at a higher rate in the comparison as opposed with intervention counties. There was no difference in the trend of oral cancer incidence at *in situ* and unknown stages between two areas, whereas distant disease (i.e., the late stage of oral cancer) occurred at a higher rate in the intervention over comparison counties.

## Discussion

During 1990–2009, the annual oral cancer incidence rates in the three Michigan counties exposed to the 2-year social marketing campaign had decreased, whereas the rate was unchanged in the comparison counties. Although there was no statistically significant slope change, the rate continued to decrease during and after the campaign only in the intervention counties. Unlike oral cancer incidence, the trend of oral cancer mortality was parallel between the two areas. Because these findings were only based on aggregate data, which are insufficient to determine whether the intervention contributed to decreasing oral cancer incidences, we explored alternative explanations and assessed how probable these were compared with the social marketing campaign.

First, the decreasing trend might be because the intervention counties, as opposed to the comparison counties, have different population profiles. Our analysis found that the key population characteristics were similar between both areas during 2000–2010. The only notable difference was a greater representation of blacks in the intervention counties, who had consistently been reported in national studies to have excess risk of developing oral cancer.^12^ During 1984–2012, US national data indicate that the oral cancer incidence rate among blacks substantially decreased, whereas a slightly increasing pattern was observed among whites.^13^ However, the reduction of the oral cancer incidence in the intervention counties was only observed when the data were only restricted to blacks, which cannot be completely explained by the national trend. The decreasing trend of the oral cancer incidence in the intervention counties in both black and white populations further supports the conclusion that other factors and perhaps the media campaign had an impact.

Second, disproportionately a large number of adults in the intervention over comparison counties might have visited dentists or modified behaviours, motivated by reasons other than our social marketing campaign. Oral cancer screening due to self-motivation or increased health insurance coverage was highly unlikely in the area that had experienced a consistent economic recession and a decreasing trend of prevalence of adults with health insurance or dental visits according to the data collected in the 2000s by Behavioral Risk Factor Surveillance System (data now shown).^14,15^ In addition, we were not aware of other large-scale oral cancer interventions in the Detroit tri-county area during 2005–2007.

Last, the observed outcome difference between the two areas might be an effect of behavioural changes or policies in the early or mid-1990s. For example, prevalence of current smokers in Michigan, which is a strong risk factor of oral cancer, decreased from 26% in 1995 to 22% in 2005, and 19% in 2010.^8,14^ However, this trend was not different between white and black Michigan residents. In addition, when broken down by stage, this decreasing trend was no longer observed. Instead, the most notable trend was an increasing rate of regional cancer in the comparison counties (4.7%), whereas the slope of the rates was almost flat in the intervention counties.

Compared with these alternative explanations, the social marketing campaign was a more probable contributor to the decreasing trend of oral cancer incidence, as supported by evidence of increased awareness of the importance of oral cancer screening, successful screening appointments and increased requests for oral cancer screening from patients as a result of the social marketing campaign.^2,3^

Like all ecological evaluation studies, this study has several limitations. First, the analysis relied on aggregate data and consequently it was difficult to assign benefits of the intervention to individuals. Second, even if we explored three alternative causes of the observed trend, we might have missed other important causes. Third, the follow-up time might not be sufficient to observe a direct impact of the campaign, which could be the reason of no statistically significant reduction of incidence rates (i.e., regression slope change) during the intervention period. Fourth, health system interventions and changes in the reporting system might influence mortality trends during the study period. Yet, due to lack of data, we were not able to account for these external factors. Last, this study finding is based on ecological data and because of lack of individual level data extrapolation is very limited and may not be valid for other population groups at the other setting.

## Conclusions

Population-level data suggested that the 2-year social marketing campaign was more likely to be associated with a trend of a greater reduction of oral cancer incidence rates in the Detroit tri-county areas versus comparison counties. Although oral cancer is a deadly disease, the awareness of this cancer has been relatively low.^9^ This study underscores the need for concerted effort to inform the public, especially in high-risk communities, of the impact of oral cancer on life and how the disease can be detected early and treated. Screening for oral cancer should be promoted as part of regular medical and dental check-ups.
