# Supplementary figures and images for: Exploring a potential impact of a social marketing campaign on reducing oral cancer incidences in Michigan: an ecological study
Source: BDJ Open. 2015 Dec 18;1:15005–. doi: 10.1038/bdjopen.2015.5 (PMC5842868; doi:10.1038/bdjopen.2015.5)

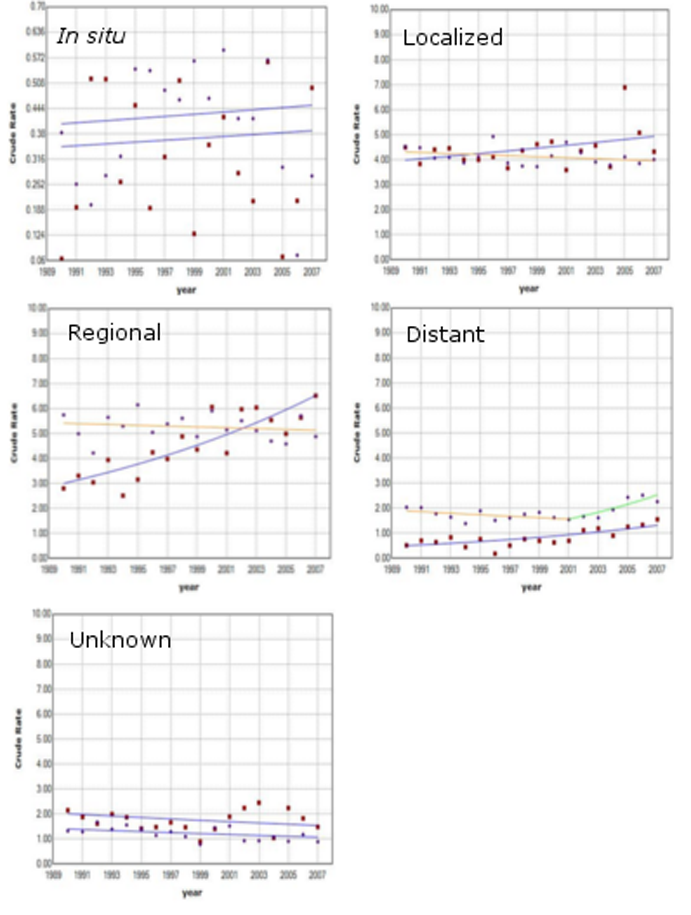

Supplement: Supplementary Figure S1 [file bdjopen20155-s1.png]
