# Supplementary material for: Exploring a potential impact of a social marketing campaign on reducing oral cancer incidences in Michigan: an ecological study
Source: BDJ Open. 2015 Dec 18;1:15005–. doi: 10.1038/bdjopen.2015.5 (PMC5842868; doi:10.1038/bdjopen.2015.5)
Supplement: Supplementary Figure legend [file bdjopen20155-s2.doc]

Figure S1. Trends of Crude Oral Cancer Incidence Rates per 100,000 Persons by Stages in the Intervention and Comparison Counties, Michigan, 1990-2007

Legend:

1. *In Situ* (early stage or early detection): square = intervention counties, star = comparision counties, blue line = a regression slope for intervention and comparison counties
2. Localized (early stage of early detection): square = intervention counties, star = comparision counties, blue line = a regression slope for intervention counties, yellow line = a regression slope for comparison counties
3. Regional (mid-stage): square = intervention counties, star = comparision counties, blue line = a regression slope for intervention counties, yellow line = a regression slope for comparison counties
4. Distant (late stage): square = intervention counties, star = comparision counties, blue line = a regression slope for intervention counties, yellow line = a regression slope for comparison counties between 1990 and 2001, green line = a regression slope for comparison counties between 2001 and 2007
5. Unknown: square = intervention counties, star = comparision counties, blue line = a regression slope for intervention and comparison counties
